# Combining MK626, a Novel DPP-4 Inhibitor, and Low-Dose Monoclonal CD3 Antibody for Stable Remission of New-Onset Diabetes in Mice

**DOI:** 10.1371/journal.pone.0107935

**Published:** 2014-09-30

**Authors:** Lei Ding, Conny A. Gysemans, Geert Stangé, Yves Heremans, Yixing Yuchi, Tatiana Takiishi, Hannelie Korf, Marie Chintinne, Richard D. Carr, Harry Heimberg, Daniel Pipeleers, Chantal Mathieu

**Affiliations:** 1 Laboratory of Clinical and Experimental Endocrinology, Campus Gasthuisberg O&N1, Faculty of Medicine, Katholieke Universiteit Leuven (KU LEUVEN), Leuven, Belgium; 2 Laboratory of Diabetes Pathology and Therapy, Diabetes Research Center, Faculty of Medicine and Pharmacy, Vrije Universiteit Brussel (VUB), Brussels, Belgium; 3 Laboratory of Beta Cell Neogenesis (BENE), Diabetes Research Center, Faculty of Medicine and Pharmacy, Vrije Universiteit Brussel (VUB), Brussels, Belgium; 4 MSD A/S, Ballerup, Denmark; University of Padova, Italy

## Abstract

Combining immune intervention with therapies that directly influence the functional state of the β-cells is an interesting strategy in type 1 diabetes cure. Dipeptidyl peptidase-4 (DPP-4) inhibitors elevate circulating levels of active incretins, which have been reported to enhance insulin secretion and synthesis, can support β-cell survival and possibly stimulate β-cell proliferation and neogenesis. In the current study, we demonstrate that the DPP-4 inhibitor MK626, which has appropriate pharmacokinetics in mice, preceded by a short-course of low-dose anti-CD3 generated durable diabetes remission in new-onset diabetic non-obese diabetic (NOD) mice. Induction of remission involved recovery of β-cell secretory function with resolution of destructive insulitis and preservation of β-cell volume/mass, along with repair of the islet angioarchitecture via SDF-1- and VEGF-dependent actions. Combination therapy temporarily reduced the CD4-to-CD8 distribution in spleen although not in pancreatic draining lymph nodes (PLN) and increased the proportion of effector/memory T cells as did anti-CD3 alone. In contrast, only combination therapy amplified Foxp3+ regulatory T cells in PLN and locally in pancreas. These findings open new opportunities for the treatment of new-onset type 1 diabetes by introducing DPP-4 inhibitors in human CD3-directed clinical trials.

## Introduction

Monoclonal anti-CD3 antibodies are presently under investigation for the treatment of autoimmune type 1 diabetes as both phase 1–2 and 2–3 randomized controlled trials demonstrated temporary preservation of stimulated C-peptide and reduced need of exogenous insulin in patients with new-onset disease [1]–[5]. Combining anti-CD3-based approaches with β-cell health-improving agents may increase the potential of the intervention, as for now only temporary preservation of remaining β-cells is observed. Many pre-clinical studies support this hypothesis and demonstrate that such combinatory strategies achieve strong synergy, both by enhancing and extending therapeutic success while minimizing toxic events as dose reduction of anti-CD3 is possible [6], [7].

Introduction of dipeptidyl peptidase-4 (DPP-4) inhibitors, which block the aminopeptidase DPP-4 and subsequently prevent the degradation of the gut-derived incretins glucagon-like peptide-1 (GLP-1) and glucose-dependent insulinotropic peptide (GIP), in immunotherapies makes sense as this class of orally-active agents not only improves β-cell function, possibly through β-cell protection and preservation [8], but also stimulates β-cell mass through β-cell replication and neogenesis [9], [10]. Considering that DPP-4 is found both as a soluble enzyme in biological fluids [11] and as a serine protease on the surface of a variety of cell types, DPP-4 inhibitors have the potential to be multi-target compounds with (metabolically) favorable effects not limited to pancreatic islet cells. DPP-4 is also known as CD26, a T-cell marker, with a co-stimulatory role in T-cell activation through an interaction with adenosine deaminase (ADA) or caveolin (on antigen-presenting cells) [12]–[14]. Of interest, type 1 diabetic patients have increased numbers of fully differentiated effector/memory CD8+ T cells expressing high levels of CD26 [15]. CD26hi cells proliferate vigorously in response to soluble antigens, secrete T helper (Th1) cytokines (e.g. IL-2, IFN-γ), and have transendothelial migration potential [16]. DPP-4/CD26 can cleave endocrine peptides [17], neuropeptides [18] and specific chemokines [19] like stromal cell-derived factor (SDF)-1 known to elicit the migration of vasculoprotective bone marrow-derived endothelial progenitor cells (EPCs)[20]. These observations imply that DDP-4 inhibitors may enhance normal glucose homeostasis via their effects on islet β-cell mass, morphology, and survival and, in addition, via several extra-pancreatic actions.

Pre-clinical studies demonstrate that DPP-4 inhibitors, alone or in combination with other drugs, can partially correct hyperglycemia in diabetic mice [9], [21]–[23], although conflicting data have also been published [24]–[26]. To date, demonstration of efficacy of DPP-4 inhibitors in human type 1 diabetes is scarce (www.clinicaltrials.gov). A first study reported decreased insulin requirements in new-onset type 1 diabetics by addition of sitagliptin to exogenous insulin therapy (NCT01235819)[27]. Similarly, several other trials are currently evaluating DPP-4 inhibitors (e.g. sitagliptin, saxagliptin, and vildagliptin) as add-on to insulin in recently-diagnosed type 1 diabetics (NCT01922817; NCT01559025; NCT01155284).

Here, we investigate whether adding DPP-4 inhibition (MK626, a DPP-4 inhibitor with appropriate pharmacokinetic properties in mice) to a subtherapeutically low dose of monoclonal CD3 antibodies reverses diabetes in new-onset diabetic NOD mice and we provide insights in the mechanism of immunomodulation and β-cell repair/expansion by the combinatory immunotherapy.

## Materials and Methods

### Animals

NOD mice, obtained from Prof. Wu (Beijing, China), were bred in our animal facility since 1989 under semi-barrier conditions. Mice were screened for glucosuria and considered diabetic if non-fasting blood glucose levels exceeded 200 mg/dl for 2 consecutive days (AccuCheck, Roche Diagnostics, Vilvoorde, Belgium)[28]. NOD-scid mice were bred from stocks purchased from the Jackson Laboratory (Bar Harbor, ME). All animal breeding and experiments were approved by the ethical committee of the Katholieke Universiteit Leuven (#087-2009).

### Reagents for animal treatment and follow up

Hamster anti-mouse CD3 mAb (clone145-2C11; BioXCell, West Lebanon, NH) and control hamster IgG (BioXCell), were administered to new-onset diabetic NOD mice at a low, subtherapeutic dose of 2.5 µg/mouse i.v. daily for 5 consecutive days as published [28]–[30]. MK626, an analog of des-fluoro-sitagliptin (Merck Research Laboratories, West Point, PA), was administered, 3 mg/kg p.o. daily, for 3 weeks after anti-CD3 administration, as this dose was shown to maximize plasma DPP-4 inhibition and increase intact (biologically active) GLP-1 levels in pharmacokinetic studies in mice [31]. Sex- and age-matched mice were randomly assigned to one of four treatment groups: 1) IgG+placebo (PBS); 2) IgG+MK626; 3) anti-CD3+placebo (PBS); and 4) anti-CD3+MK626. Weight and glycemia were measured 3 times weekly. Diabetes remission was defined as absence of glycosuria and hyperglycemia (<200 mg/dl) on two consecutive days. Serum, spleen, pancreatic draining lymph nodes (PLN) and pancreas were harvested for analyses 2 and/or 7 weeks after treatment discontinuation.

### Immune monitoring

Single cell suspensions were prepared from spleen and PLN and stained with fluorescently-labeled antibodies against CD3e (145-2C11), CD4 (GK1.5), CD8a (53–6.7), CD25 (PC61), CD26 (H194-112), CD44 (IM7), CD62L (MEL-14), CD69 (H1.2F3) and matching isotype controls (all eBioscience, San Diego, CA). FoxP3 staining was performed using FoxP3 staining kit (eBioscience). Cells were acquired on a Gallios flow cytometer and data were analyzed with Kaluza software (Beckman Coulter, Suarlée, Belgium).

### β- and α-cell function

Mice were fasted for 12 hours, injected intraperitoneally with D-glucose (2 g/kg body weight) and glycemia was measured after 15 minutes. Insulin, C-peptide, and glucagon levels in sera were measured with mouse insulin ELISA (Mercodia, Uppsala, Sweden), rat/mouse C-peptide 2 ELISA (Merck Millipore, Billerica, MA), and glucagon EIA (Gentaur, London, UK) kits. Pancreases were harvested for histological analyses (2 weeks after therapy), RNA isolation (7 weeks after therapy) and/or for insulin content determination (2 and 7 weeks after therapy) after acid/ethanol extraction as described [29].

### Histology and immunofluorescent microscopy

After weighing, head and tail sections of pancreata were fixed overnight (4% formalin), paraffin-embedded and sectioned entirely.

#### Insulitis grading

Pancreatic sections were stained with hematoxylin and eosin. Islets were observed under light microscopy and graded by an individual blinded to the experimental design. At least 25 islets per pancreatic sample were scored for islet infiltration as follows: 0, no infiltration; 1, peri-insulitis; 2, islets with lymphocyte infiltration in less than 50% of the area; 3, islets with lymphocyte infiltration in more than 50%; 4, islets that are completely destroyed.

#### Total β- and α-cell volume

Total β- and α-cell volume were determined in 4-µm thick insulin or glucagon-stained sections, 36 µm apart, spanning the whole pancreatic tissue by the Cavalieri principle and expressed as µl per pancreas [32].

#### CldU and/or IdU labeling

Proliferating cells were labeled during 24 hours by sequentially administering thymidine analogs 5-chloro-2′-deoxyuridine (CldU, Sigma) and 5-iodo-2′-deoxyuridine (IdU, Sigma) in drinking water at 1 mg/ml. Staining of thymidine analogs was done as described [33]. At least 20 islets were analyzed per animal. Images were acquired with a Zeiss Axioskop 2 (Carl Zeiss, Jena, Germany) and captured with an Orca-R2 digital camera (Hamamatsu, Hamamatsu City, Japan). CldU+ and IdU+ single as well as CldU+IdU+ double-labeled insulin+ β-cell ratios were calculated as means ± s.e.m., with at least 5,000 β-cells counted per experimental group.

#### β-cell regranulation and replication

Insulin and glucose transporter (GLUT)-2 were detected with guinea pig anti-insulin (in-house, 1∶5,000) and rabbit anti-GLUT2 (Alpha Diagnostic, San Antonio, TX, 1∶200, heat-mediated antigen retrieval with 10 mM citrate pH 6.0). Secondary antibodies were Cy3- or Alexa Fluor 488-conjugated. Nuclei were stained with Hoechst 33342 (Sigma). The percentage of islets containing GLUT2+insulin- or GLUT2+insulin+ cells was determined on an average of 80 islets per sample.

Additionally, sections were stained with mouse anti-insulin (Sigma) and rabbit-anti-Ki67 (Novocastra, Leica Biosystems, Newcastle, UK), both at 1∶2,000 and detected with Alexa Fluor 488-conjugated anti-mouse and Alexa Fluor 594-conjugated anti-rabbit IgG (Life Technologies). The percentage of insulin+Ki67+ β-cells was counted in an average of 5,000 β-cells per sample.

#### Islet vasculature

Sections were stained with mouse anti-insulin (Sigma) and rabbit anti-collagen type IV (Col IV) (1∶80, Merck Millipore). Secondary antibodies were Alexa Fluor 488-conjugated anti-mouse and Alexa Fluor 594-conjugated anti-rabbit IgG (Life Technologies). The area of Col IV+ vessels within an islet was measured in at least 80 islets from each experimental group. All images were analyzed using ImageJ (NIH, Bethesda, MD).

#### RT-qPCR

mRNA amounts of mouse CD31, collagen (Col) IVα1, FoxP3, GLUT-2, insulin (INS)-2, pancreatic and duodenal homeobox (PDX)-1, NeuroD, SDF-1, and vascular endothelial growth factor (VEGF) were quantified as described and normalized to β-actin and the 60S ribosomal protein L27 (RPL27)[34]. Primers, probes and reaction conditions are available on request.

### Statistical analysis

Differences in diabetes incidence were assessed using the Mantel-Cox Log-rank test. Statistical significance of other comparisons was tested using Student's *t*-test or ANOVA (data with normal distribution) or non-parametric Mann-Whitney *t*-test or Kruskal-Wallis test. Graphs were plotted and statistics calculated with GraphPad Prism software (La Jolla, San Diego, CA). p<0.05 was considered significant.

## Results

### MK626 enhanced anti-CD3-induced remission of diabetes in new-onset diabetic NOD mice

**Figure 1:**
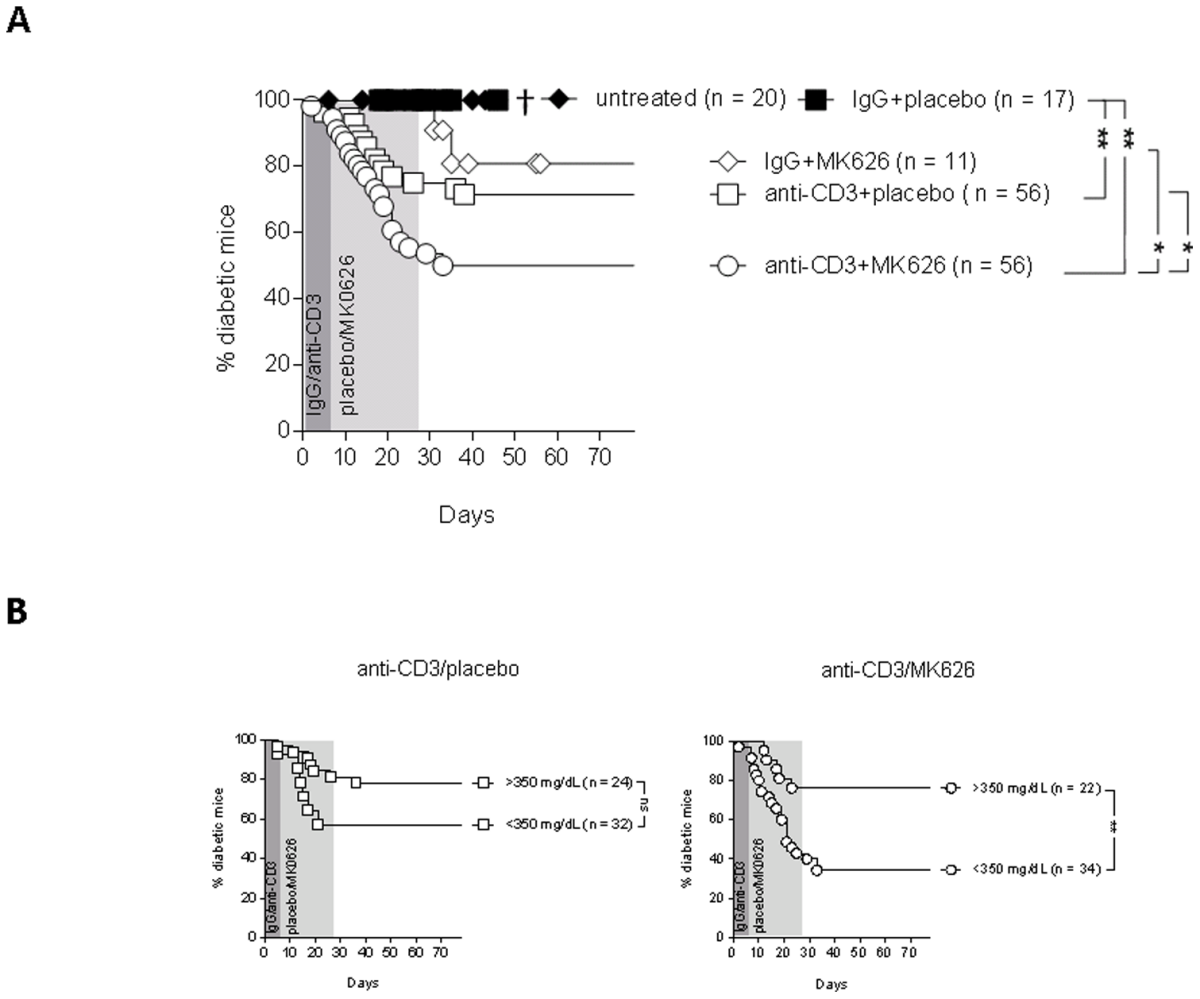
Anti-CD3+MK626 treatment induced stable diabetes reversal in new-onset diabetic NOD mice. New-onset diabetic NOD mice (2 consecutive readings of blood glucose values>200 mg/dl), received treatments as indicated in figures. Random blood glucose values were monitored until 7 weeks after therapy. The number of mice assigned to each treatment group is shown in the figure. The gray area represents the treatment period. (A) Percentage of diabetic mice per treatment group. † indicates dead or moribund mice. (B) Therapeutic efficacy of anti-CD3+placebo (left) and anti-CD3+MK626 treated (right) NOD mice was stratified according to blood glucose values at diabetes diagnosis. * p<0.05, ** p<0.01.

Here, we studied whether combining a short-course low-dose anti-CD3 regimen with MK626 (a novel DPP-4 inhibitor) improved diabetes reversal in new-onset diabetic NOD mice. No difference in starting blood glucose values was present between treatment groups (Fig. S1A). Remission of diabetes was not achieved in untreated (n = 20) or in IgG+placebo treated mice (n = 17), eventually resulting in wasting (mean survival time of 34±12 and 28±7 days after disease onset for untreated and IgG+placebo treated controls respectively)(Fig. 1A). In line with our previous observations [29], [30], a short course of low-dose anti-CD3 (clone 145-2C11; 2.5 µg/day from day 0–4) corrected diabetes in 27% of new-onset diabetic mice (n = 56, p<0.01 vs. controls). Administration of IgG+MK626 normalized hyperglycemia in only 20% of mice (n = 11), whereas combining anti-CD3 with MK626 resulted in diabetes reversal in 50% of treated mice (n = 56; p<0.01 vs. controls; p<0.05 vs. IgG+MK626 and anti-CD3+PBS treated groups) (Fig. 1A). This effect was durable, lasting at least 7 weeks after therapy (Fig. S1B). Progressive reversal of hyperglycemia started between 7 and 33 days after treatment initiation and the average blood glucose level in cured mice was reduced from 332 mg/dl, prior to treatment, to 142 mg/dl (Fig. S1A and B). No signs of toxicity related to the treatment with anti-CD3+MK626 (weight loss, altered behavior, fur loss, urine and stool discoloration) were observed (Fig. S1C).

Given the higher rate of disease remission observed with anti-CD3+MK626 immunotherapy, we examined how this observation related to the blood glucose values at the time of disease onset. Anti-CD3 combined with MK626 stably reverted 66% of new-onset diabetic mice with starting blood glucose levels≤350 mg/dl (n = 34), in contrast to 43% of mice treated with anti-CD3+placebo (n = 32) (p<0.001, Fig. 1B). In mice with the highest levels of blood glucose (≥350 mg/dl) at start of therapy, the rate of diabetes correction was comparable between anti-CD3+MK626 and anti-CD3+placebo treated groups (23% (n = 22) and 22% (n = 24) respectively) (Fig. 1B).

### Combination therapy of anti-CD3 with MK626 expanded the CD4+CD25+FoxP3+ Treg compartment

**Figure 2:**
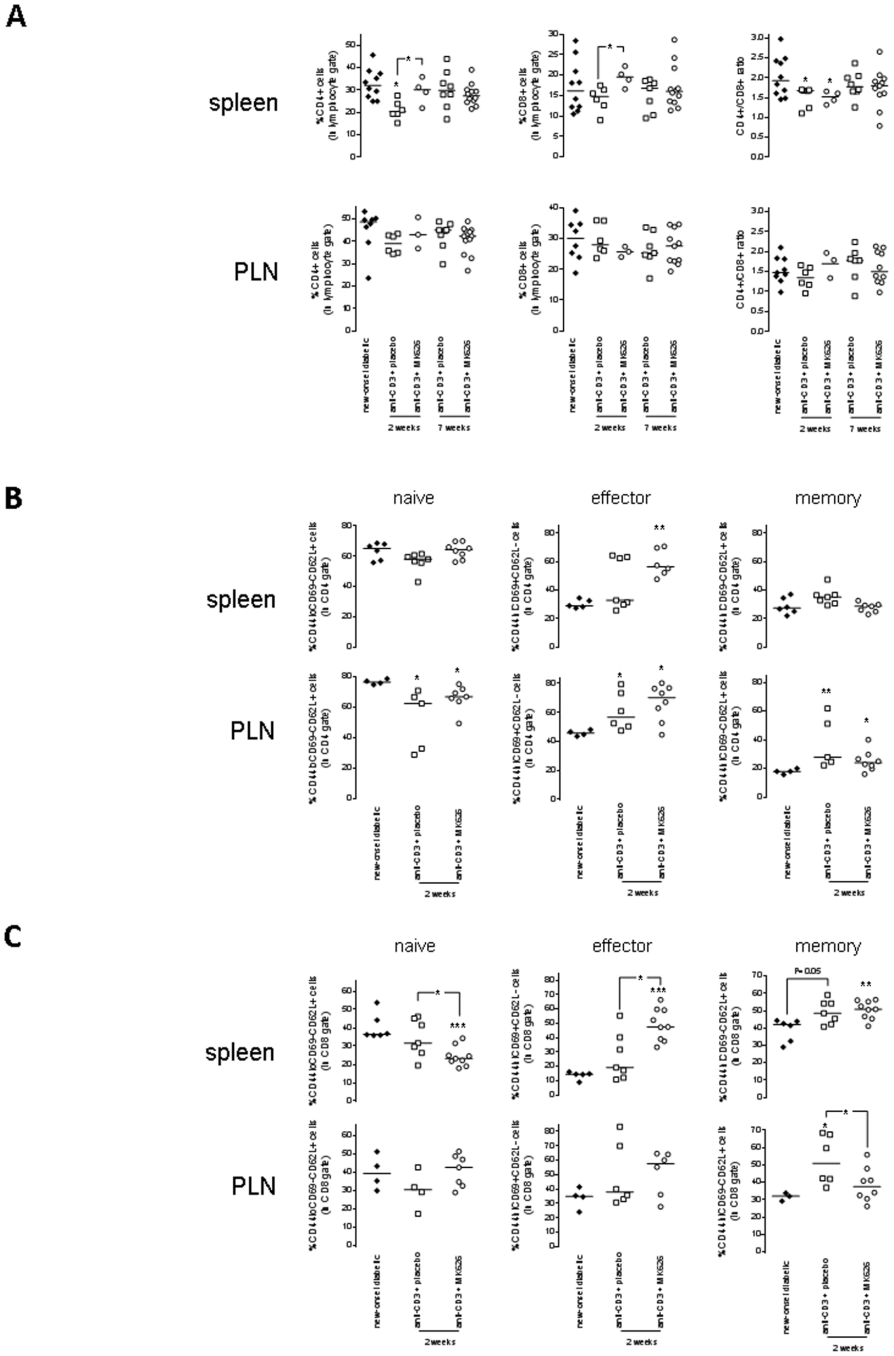
Anti-CD3+MK626 treatment temporarily reduced the CD4-to-CD8 distribution and enlarged the proportion of effector/memory T cells. (A) Frequency of CD4+ T cells (left panel) and CD8+ T cells (middle panel) as well as CD4-to-CD8 ratio (right panel) in spleens and PLN of new-onset diabetic NOD mice that remained protected for 2 and 7 weeks following each of the treatments. (B) Frequency of naïve (CD44loCD69-CD62L+), effector (CD44hiCD69+CD62L-) and memory (CD44hiCD69-CD62L+) T cell subsets in CD4+ (upper) and CD8+ (lower) gate from spleens and PLN of new-onset diabetic NOD mice that remained protected for 2 and 7 weeks following each of the treatments. Each dot represents an individual mouse. * vs. new-onset diabetic mice. One symbol p<0.05; two symbols p<0.01; three symbols p<0.001.

Compared to untreated new-onset diabetic NOD mice, both anti-CD3+placebo and anti-CD3+MK626 decreased the proportion of CD4+ T cells in the spleen 2 weeks after therapy, consequently reducing the CD4-to-CD8 ratio, although no equivalent decrease was observed in PLN (Fig. 2A). The proportion of CD8+ T cells remained largely unchanged. Importantly, CD4+ T-cell frequencies were restored to pretreatment levels 7 weeks after therapy. Before this restoration, the anti-CD3-based approaches increased the frequency of effector (CD44hiCD69+CD62L-) and/or memory (CD44hiCD69-CD62L+) CD4+ and CD8+ T cells in the spleen and/or PLN (Fig. 2B and C), possibly as a results of homeostatic proliferation. Of note, these activated/memory T cells displayed a CD26hi phenotype (Fig. S2A and B)[35].

**Figure 3:**
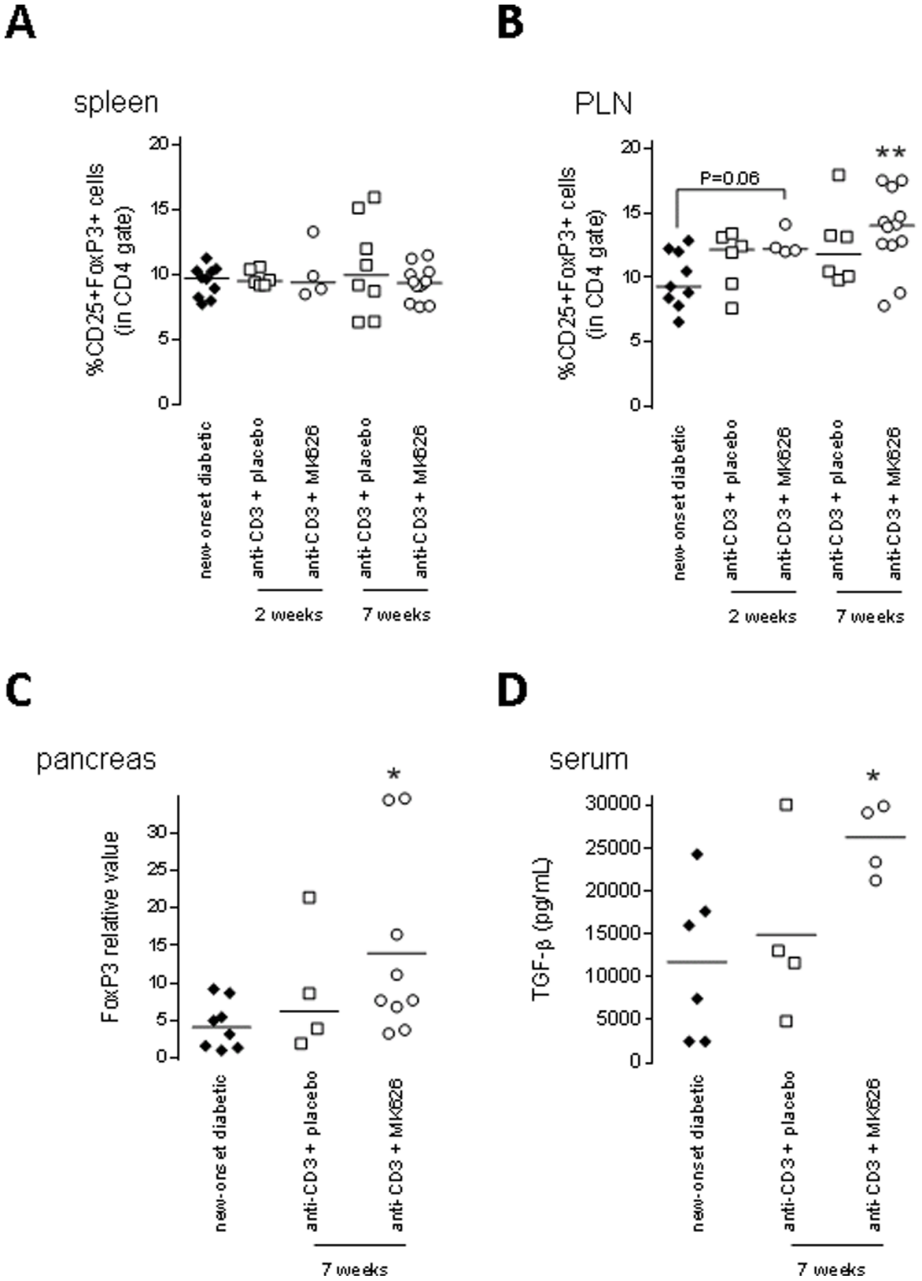
Bias toward a regulatory phenotype in anti-CD3+MK626 treated NOD mice. *A* and *B*: Treg (CD4+CD25+FoxP3+) frequencies were determined by FACS in spleens (A) and PLN (B) of new-onset diabetic NOD mice that remained protected for 2 and 7 weeks following each of the treatments (upper panels). (C) Quantitative PCR analysis for FoxP3 expression in pancreatic tissue of the mice treated with anti-CD3+placebo or anti-CD3+MK626 7 weeks after therapy as well as untreated new-onset controls. (D) Serum TGF-β1 levels for each treatment group 7 weeks after treatment period (lower panels). Each dot represents an individual mouse. * *p*<0.05, ** *p*<0.01 vs. new-onset diabetic NOD mice.

CD4+CD25+FoxP3+ T-cells, considered as regulatory T cells (Tregs), were increased in PLN 7 weeks after therapy when compared with untreated new-onset diabetic controls (p<0.01) (Fig. 3A and B). This phenomenon was already visible 2 weeks after therapy with a trend to higher frequencies of CD4+CD25+FoxP3+ Tregs in the PLN (p = 0.06 vs. untreated controls) (Fig. 3B). Interestingly, FoxP3-expressing Tregs had absent or low expression of CD26 (Fig. S2C). No alterations in CD4+CD25+FoxP3+ Tregs were observed in spleen, whereas the relative mRNA expression of FoxP3 was significantly increased in pancreases of anti-CD3+MK626 treated NOD mice (p<0.05 vs. untreated controls) (Fig. 3C).

As transforming growth factor (TGF)-β can promote the expansion and maintenance of Tregs [36]; we measured TGF-β levels in the sera of cured mice 7 weeks after therapy. Importantly, we observed a significant increase in TGF-β production in serum of anti-CD3+MK626 group (p<0.05 vs. untreated controls) (Fig. 3D).

### Combination therapy of anti-CD3 with MK626 gradually induced recovery of β-cell secretory function with resolution of severe insulitis and preservation of β-cell volume/mass

**Figure 4:**
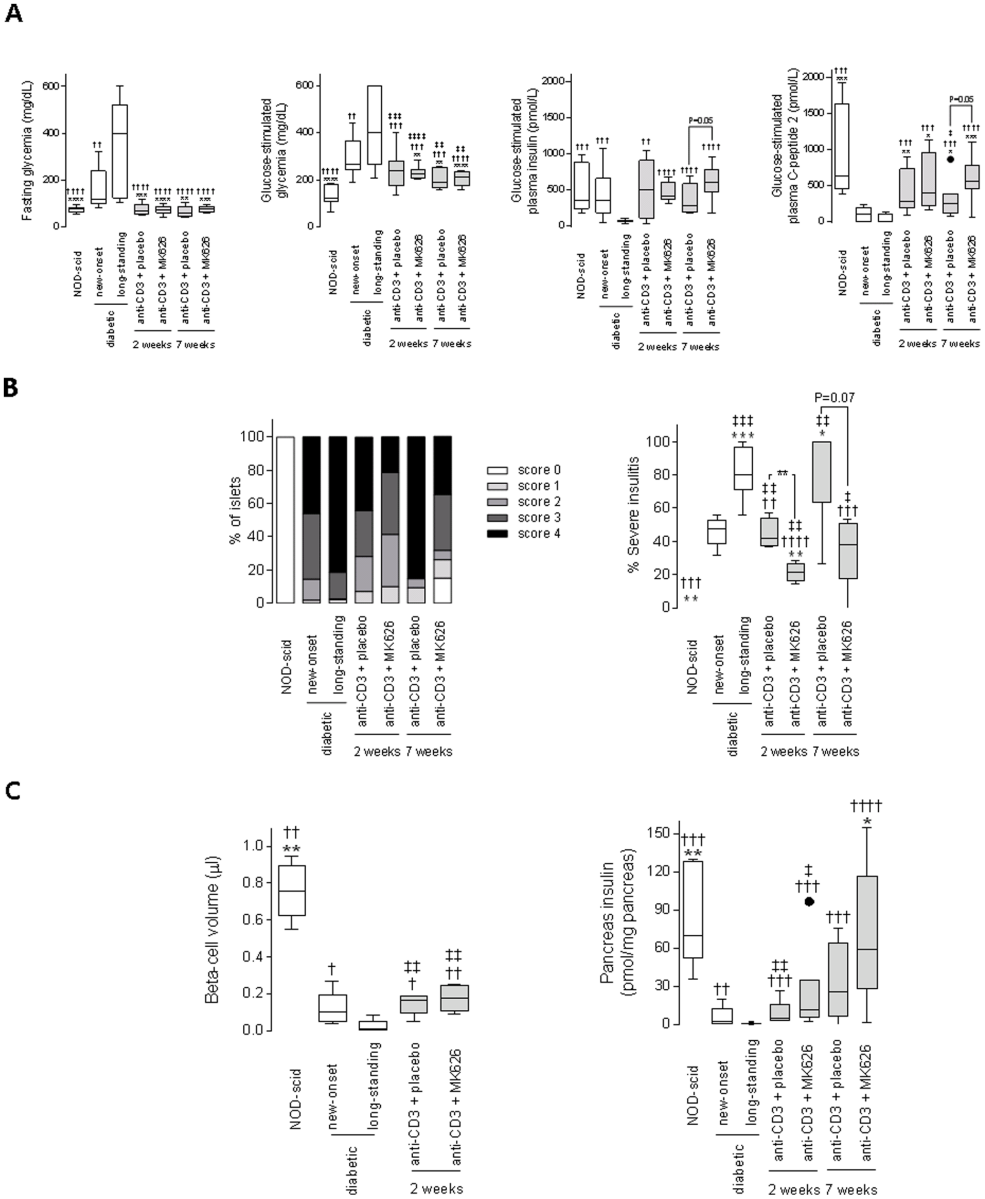
Anti-CD3+MK626 treatment rescued islet β-cell function, resolved destructive insulitis and preserved β-cell volume/mass in new-onset diabetic NOD mice. Mice treated as indicated were fasted and then challenged with glucose load for 15 minutes. (A–C) Changes in fasting and glucose-stimulated blood glucose values, glucose-stimulated plasma insulin and C-peptide levels (A), severity of insulitis and destructive lesions (B), pancreatic β-cell volume (µl) and insulin content (pmol/mg pancreas)(C) for the control groups and anti-CD3+placebo and anti-CD3+MK626 treated mice at 2 and 7 weeks after therapy. Pancreas sections were classified as with no (score 0), peri- (score 1), <50% (score 2), >50% (score 3) and destructive (score 4) insulitis. Only animals that normalized their blood glucose (<200 mg/dl) during reversal of diabetes by anti-CD3+placebo or anti-CD3+MK626 treatment are shown in the figure. All box plots are depicted with Tukey whiskers from minimum to maximum (values from at least 5 to 16 animals per group). * vs. new-onset diabetic mice; † vs. longstanding diabetic mice; ‡ vs. age-matched normoglycemic NOD-scid mice. One symbol *p*<0.05; two symbols *p*<0.01; three symbols *p*<0.001 and four symbols *p*<0.0001.

At 2 and 7 weeks after stopping therapy, NOD mice free of diabetes after anti-CD3+placebo and anti-CD3+MK626 treatment had almost identical fasting blood glucose concentrations as age-matched normoglycemic NOD-scid mice (Fig. 4A). However, upon glucose challenge, these mice showed glucose intolerance when compared with age-matched normoglycemic NOD-scid mice. Interestingly, glucose control gradually improved after diabetes reversal by anti-CD3+MK626 treatment, in spite of the cessation of anti-CD3+MK626 intervention. Although not statistically significant, both glucose-stimulated insulin and C-peptide concentrations were higher in mice cured by anti-CD3+MK626 compared to mice cured by anti-CD3+placebo 7 weeks after therapy (Fig. 4A), and comparable to age-matched normoglycemic NOD-scid mice.

Histological analysis conducted 2 weeks after stopping therapy revealed that cured anti-CD3+MK626 treated NOD mice had significantly less islets with destructive insulitis compared to diabetic mice either at the time of disease onset or several weeks after onset (21% vs. 46%, p<0.01 and 82%, p<0.0001 respectively) (Fig. 4B). Moreover, severity of insulitis in cured anti-CD3+MK626 treated mice was reduced compared with cured anti-CD3+placebo treated mice (44%, p<0.01) (Fig. 4B). While islet infiltration was increased over time in untreated diabetic controls and in anti-CD3+placebo treated mice, destructive insulitis decreased in most anti-CD3+MK626 treated mice (35% at 7 weeks after treatment discontinuation).

Quantification of β- and α-cell volume by immunostaining on pancreata revealed that at diabetes diagnosis, only few insulin-secreting β-cells remained in the residual islet remnants, and pancreata consisted mainly of α-cells with total β-cell volume (0.12±0.04 µl per pancreas) being only 15% of that found in age-matched normoglycemic NOD-scid mice (0.76±0.07 µl per pancreas). In contrast to control diabetic mice that showed a further decrease of β-cell volume (0.03±0.02 µl per pancreas), mice cured either by anti-CD3+placebo or anti-CD3+MK626 therapy could retain their residual β-cell volume (0.15±0.03 and 0.18±0.03 µl per pancreas)(Fig. 4C). However, total β-cell volume remained clearly below the volume found in age-matched normoglycemic NOD-scid (control) mice. Pancreatic α-cell volume was stable after each of the anti-CD3-based therapies compared to new-onset diabetic controls (data not shown).

**Figure 5:**
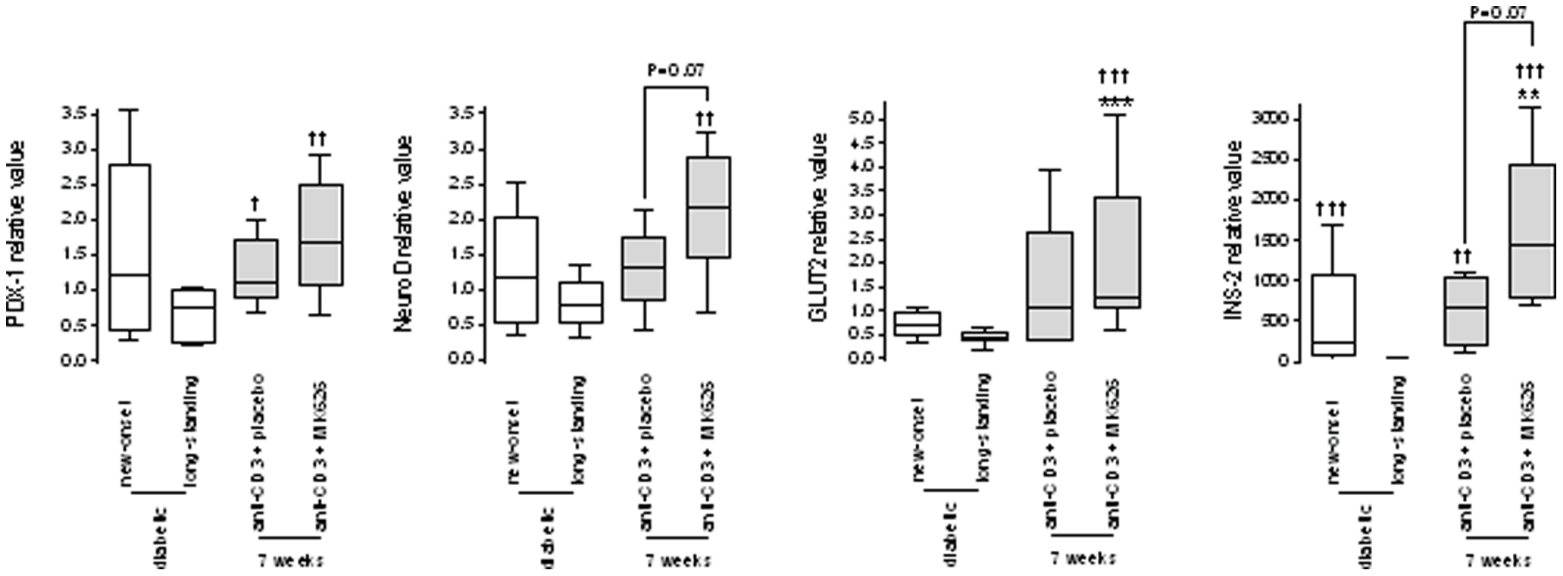
Anti-CD3+MK626 treatment stimulated expression of endocrine β-cell markers in new-onset diabetic NOD mice. PDX-1, NeuroD, GLUT2 and INS-2 gene expression locally in pancreata of untreated new-onset and long-standing diabetic controls as well as in those retrieved from mice 7 weeks after anti-CD3+placebo or anti-CD3+MK626 therapy. * vs. new-onset diabetic mice; † vs. longstanding diabetic mice. One symbol p<0.05.

Insulin content in pancreata of cured anti-CD3+placebo or anti-CD3+MK626 treated mice 2 weeks after treatment discontinuation was comparable with that of new-onset diabetic mice (Fig. 4C). Remarkably, 7 weeks after stopping therapy, pancreata of anti-CD3+MK626 treated mice had a distinctly higher insulin content (67.72±18.44 pmol/mg pancreas) compared with diabetic mice (5.79±3.55 pmol/mg pancreas; p<0.05 vs. new-onset and 0.17±0.10 pmol/mg pancreas; p<0.0001 vs. longstanding) (Fig. 4C) and not significantly different from age-matched normoglycemic NOD-scid mice (86.15±18.28 pmol/mg pancreas; Fig. 4C). These data were corroborated by the 3-fold increased mRNA expression of INS-2 in the pancreata of cured anti-CD3+MK626 treated mice 7 weeks after therapy compared to those of cured anti-CD3+placebo treated mice (p = 0.07) (Fig. 5). Other endocrine mRNAs, such as PDX-1, NeuroD, and GLUT2, were also readily detected in the pancreata of cured anti-CD3+MK626 treated mice (Fig. 5).

### Combination therapy of anti-CD3 with MK626 induced β-cell regranulation with little proliferation

**Figure 6:**
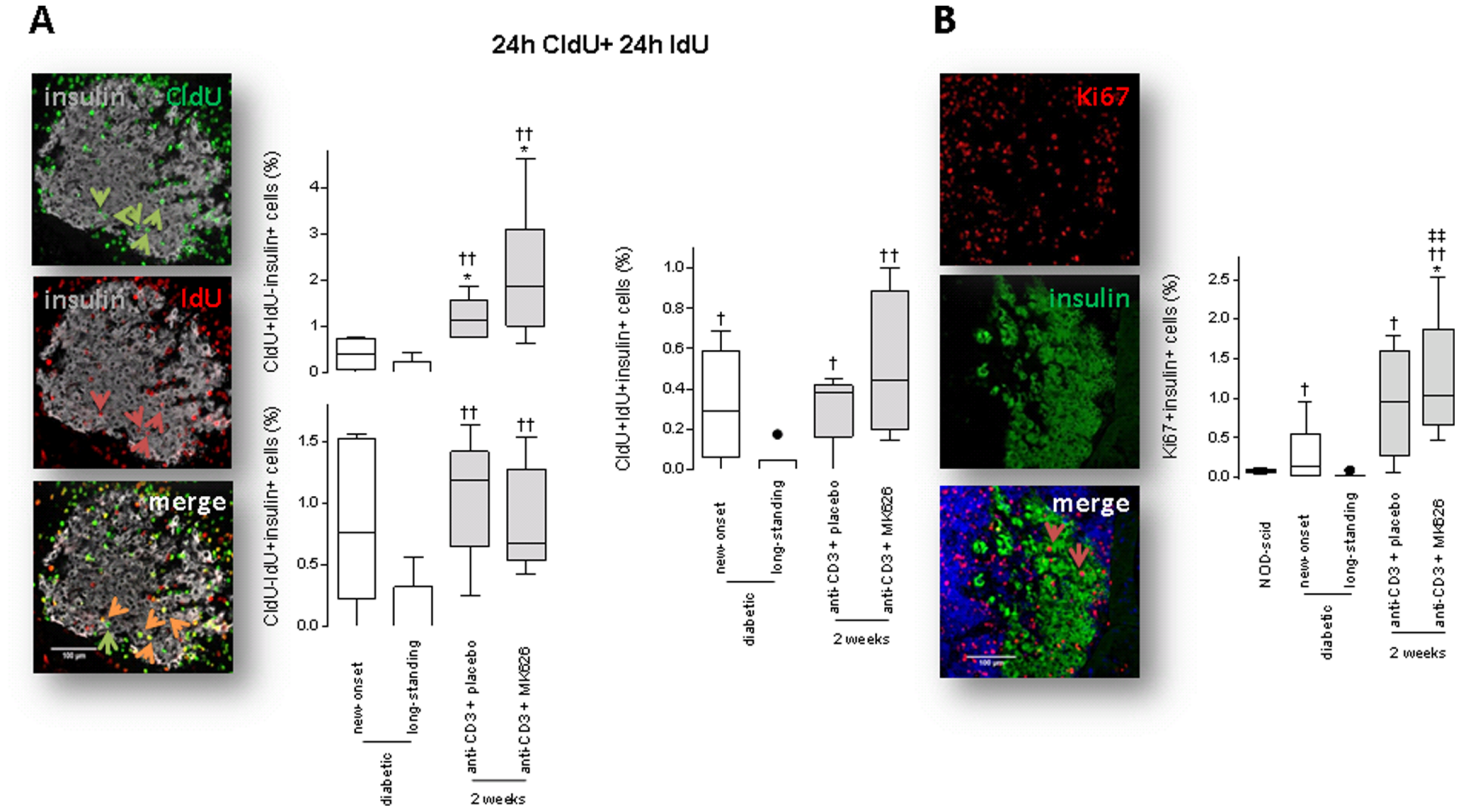
Anti-CD3+MK626 treatment allowed marginal β-cell replication in new-onset diabetic NOD mice. (A) Confocal microscopy images of insulin (gray), CldU (green), IdU (red) (left). Green arrowheads indicate CldU+ positive β-cells, red arrowheads indicated IdU+ positive β-cells and orange arrowheads indicate CldU+IdU+ β-cells. Quantitative analysis of β-cell proliferation after thymidine analog labeling, as measured by insulin+ cells that co-stained for CldU+, IdU+, or both (right). Box plots are depicted with Tukey whiskers from minimum and maximum. (B) Representative images from confocal microscopy of Ki67 (red) and insulin (green) staining of pancreatic sections from anti-CD3+MK626 treated (2 weeks after therapy) mice. Arrow heads indicate Ki67+ β-cells. The box plots represent the percentage of insulin+Ki67+ cells in at least 80 islets and are depicted with Tukey whiskers from minimum and maximum. For each figure set, at least 5,000 insulin+ cells were counted from 20 non-serial sections for the control and treated mice (5 to 6 per group), respectively. * vs. new-onset diabetic mice; † vs. longstanding diabetic mice. One symbol p<0.05; two symbols p<0.01.

To allow tracing of (re)-replicating β-cells in vivo, we labeled cured mice 2 weeks after therapy and untreated diabetic controls with the thymidine analogs CldU (for 24 hours) and then with IdU (for 24 hours) followed by immediate killing of the mice. Pancreata were processed for immunofluorescent staining for insulin and thymidine analogs. At the end of the sequential labeling period, only a very small amount of β-cells had re-replicated as indicated by CldU+IdU+ β-cells, while slightly higher numbers of CldU+IdU- and CldU-IdU+ β-cells were found (Fig. 6A). Anti-CD3+MK626 as well as anti-CD3+placebo were equally potent in inducing an enhanced, however minor, proliferation frequency in β-cells from cured mice compared with β-cells from untreated diabetic mice (either at onset or several weeks after) (Fig. 6A).

The proliferation marker Ki67 was also measured in the aforementioned experimental groups. The percentage of insulin+ β-cells expressing Ki67 was 0.25±0.18% in pancreata of new-onset diabetic NOD mice and greatly decreased with diabetes duration (0.014±0.014% in pancreases from long-standing diabetics) (Fig. 6B). Two weeks after anti-CD3+MK626 treatment, the proportion of insulin+Ki67+ β-cells in the islets was increased to 1.22±0.35% in pancreata of cured mice (p<0.05 vs. new-onset, p<0.01 vs. longstanding diabetics and p<0.01 vs. age-matched normoglycemic NOD-scid mice, Fig. 6B) but not statistically different to values found in pancreata from mice cured by anti-CD3+placebo therapy (0.94±0.31%).

**Figure 7:**
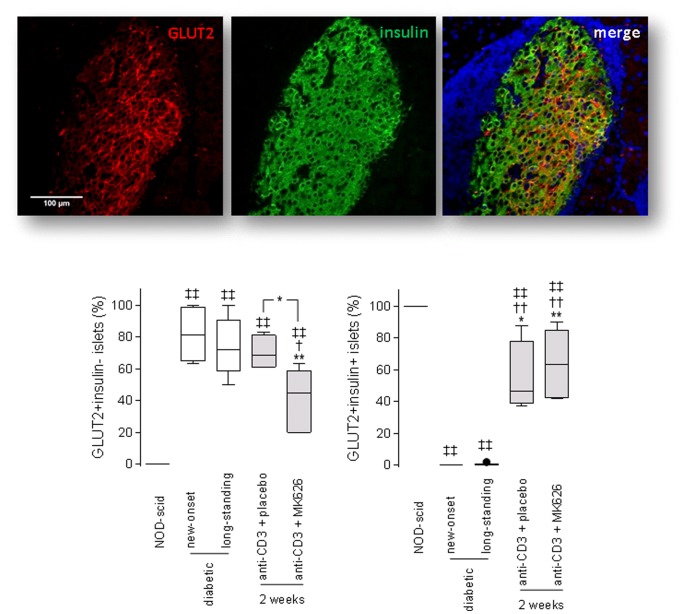
Anti-CD3+MK626 treatment rejuvenated pancreatic islets from new-onset diabetic NOD mice. Confocal microscopy images of GLUT-2 (red) and insulin (green) staining of pancreatic sections from anti-CD3+MK626 treated mice. The box plots represent the percentage of GLUT-2+insulin- (right) and GLUT-2+insulin+ (left) pancreatic islets from mice treated with anti-CD3+placebo or anti-CD3+MK626 2 weeks after therapy stop as well as untreated new-onset and longstanding diabetic controls and are depicted with Tukey whiskers from minimum and maximum. Islets (at least 80) from each experimental group (5 to 7 mice) from 2 to 4 non-serial sections per mouse were examined. * vs. new-onset diabetic mice; † vs. longstanding diabetic mice; ‡ vs. age-matched normoglycemic NOD-scid mice. One symbol p<0.05; two symbols p<0.01.

We also studied whether β-cells that were degranulated (i.e. depleted of secretory granules), yet not fully destroyed, recovered after anti-CD3+MK626 therapy. Confirming others' previous observations [7], the majority of pancreatic islets of untreated diabetic controls (both new-onset and longstanding) contain GLUT-2+ but insulin- β-cells (Fig. 7). Two weeks after therapy, we counted significantly less islets with insulin-GLUT-2+ β-cells in the cured anti-CD3+MK626 group (41.6±7.9%) compared to diabetic controls (81.8±7.6%; p<0.01 vs. new-onset and 74.2±8.2%; p<0.02 vs. longstanding) and to cured anti-CD3+placebo group (70.3±5.3%; p<0.05) but percentages were not restored to those found in age-matched normoglycemic NOD-scid (control) mice. Moreover, the amount of islets with insulin+GLUT-2+ β-cells markedly increased by each of the anti-CD3-based therapies compared to untreated animals (Fig. 7). Pancreatic GLUT-2 mRNA expression correlated very well with the histology findings, as demonstrated by the increased values found in cured mice after anti-CD3+MK626 therapy compared to diabetic controls (Fig. 5).

### Combination therapy of anti-CD3 with MK626 induced restoration of the islet angioarchitecture via SDF-1- and VEGF-dependent actions

**Figure 8:**
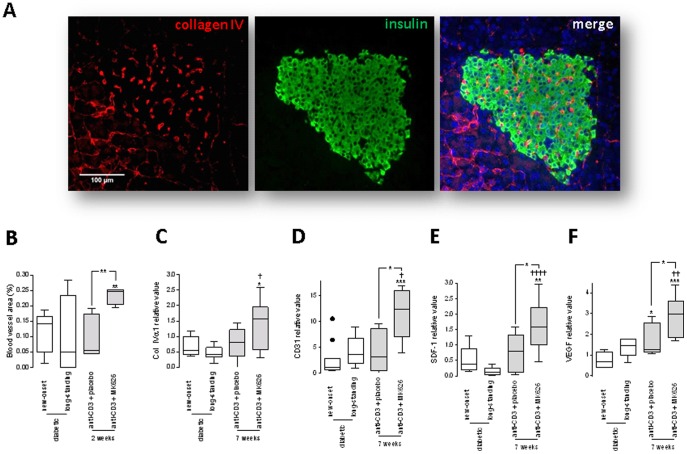
Anti-CD3+MK626 treatment promoted islet vascularity through SDF-1 and VEGF-dependent pathways. (A) Fluorescence microscopy images of collagen IV (red) and insulin (green) immunostaining of pancreatic sections from mice given anti-CD3+MK626 treatment. (B) Quantification of the area occupied by the blood vessels in pancreatic islets from mice treated with anti-CD3+placebo or anti-CD3+MK626 2 weeks after therapy as well as untreated new-onset and longstanding diabetic controls. Islets (at least 80) from each experimental group (5 to 6 mice) from 2 to 4 non-serial sections per mouse were examined. (C–F) Quantitative PCR analysis for Col IV1α1 (C), CD31 (D), SDF-1 (E), and VEGF (F) expression in pancreatic tissue of the mice treated with anti-CD3+placebo or anti-CD3+MK626 7 weeks after therapy as well as untreated new-onset and longstanding diabetic controls (8 to 11 mice per group). Box plots are depicted with Tukey whiskers from minimum and maximum. * vs. new-onset diabetic mice; † vs. longstanding diabetic mice. One symbol p<0.05; two symbols p<0.01; three symbols p<0.001 and four symbols p<0.0001.

Col IV α1 and α2 are found primarily in the basal lamina, and are essential components for the maintenance of integrity and function of basement membranes. We found that 2 weeks after therapy anti-CD3+MK626 treatment reconstituted the intra-islet capillaries (Fig. 8A) as indicated by increased pancreatic blood vessel area (0.23±0.01%) in cured mice compared to new-onset diabetic mice (0.11±0.03%; p<0.01) and to cured anti-CD3+placebo treated mice (0.10±0.03%; p<0.01) (Fig. 8B). Anti-CD3+MK626 also increased the mRNA levels of Col IVα1 (Fig. 8C) as well as of the endothelial cell marker CD31 (Fig. 8D) in pancreata from cured anti-CD3+MK626 treated mice 7 weeks after therapy compared to those from new-onset diabetic mice and from cured anti-CD3+placebo treated mice. As SDF-1 and VEGF are involved in endothelial remodeling and repair [37], we tested whether anti-CD3+MK626 had a differential effect on SDF-1 and VEGF expression locally in the entire pancreas. Anti-CD3+MK626 controlled SDF-1 and VEGF mRNA levels in pancreata of cured mice 7 weeks after therapy (Fig. 8E and F), both were significantly increased compared to levels found in those of new-onset (p<0.01 and p<0.001 respectively) and longstanding diabetic mice (p<0.001 and p<0.01 respectively) and of cured anti-CD3+placebo treated mice (p<0.05).

## Discussion

Pre-clinical [29], [38] and clinical [1], [2], [4], [5] studies point towards anti-CD3 monoclonal antibodies as the most promising intervention in newly-diagnosed mice and humans with type 1 diabetes. Particularly interesting are the pre-clinical studies indicating that when anti-CD3 is combined with antigen-specific interventions, an antigen-specific restoration of tolerance is observed [29], [30], [39]. A potential weakness of these immunotherapies is that they result in an arrest of β-cell function, however at a stage where the subject was when therapy was initiated. Some pre-clinical data indicate that the positive effect of some interventions on the severity of infiltrative insulitis results in some regranulation and functional enhancement of the β-cells [40].

We show that combining a low, subtherapeutic dose of anti-CD3 with MK626 – a novel, orally active, and selective DPP-4 inhibitor (with optimized pharmacodynamics and kinetic profile in rodents) – suppressed β-cell destruction (i.e. alleviation of destructive insulitis possibly via induction of FoxP3+Tregs in PLN and locally in pancreas), combined with regranulation of the majority of the β-cells, but, more importantly, also an improvement of islet vasculature. In contrast to previous studies, where the DPP-4 inhibitor sitagliptin alone or in combination with an activating G-protein coupled receptor 119 (GPR119) clearly stimulated β-cell replication and neogenesis from cells lining the pancreatic ducts [41], in the present study; we could not observe an increased proliferation rate by our combination therapy compared to anti-CD3 alone. We speculate that this could have been a ‘false negative’ result with MK626, despite its ability to inhibit DPP-4 activity [31], as it is more rapidly metabolized in rodents, so exposure to the active drug may have been too low to see an effect. Moreover, only very high doses of sitagliptin (20 to 200 times higher compared to the current MK626 dosage) were shown to increase β-cell proliferation or neogenesis via the maintenance of high GLP-1 or GIP levels through DPP-4 inhibition, which can activate the phosphoinositol 3-kinase (PI3K), cyclic AMP (cAMP) and protein kinase A (PKA) signaling and cause cyclin D1/D2 activation [42].

The observation of changes in islet angioarchitecture is novel and occurred only in the combination of anti-CD3+MK626. The exact mechanism of induction is unclear, but a link with the immune effects of this combination therapy may be present; the increased systemic levels of the immunomodulatory cytokine TGF-β induced particularly by anti-CD3+MK626 therapy might signal directly for the observed up-regulation of FoxP3 in Tregs [43]. Several inhibitory molecules or naturally occurring ligands of CD26/DPP-4 stimulate the secretion of TGF-β [44], [45]. Interestingly, we found that anti-CD3+MK626 therapy-induced FoxP3+ Tregs were characterized by absent or low levels of surface CD26 molecules. This observation points towards a connection between CD26 and a (functionally-active) Treg phenotype. A low level of CD26 might diminish the proportion of ADA on Tregs [46]. On the other hand, a CD26 absent or low phenotype in Tregs may help to keep CD86 expression on antigen-presenting cells decreased, leading indirectly to anergy or apoptosis of antigen-experienced naïve T cells [14]. TGF-β can also activate the recruitment of VEGF-producing hematopoietic cells, creating a potent signaling network in the inflammatory milieu, which concurrently promotes neovascularization [47]. The improved islet vascularization, as evidenced by a larger intra-islet blood vessel area and increased expression levels of Col IVα1 and CD31 locally in the whole pancreas, after anti-CD3+MK626 combination therapy likely further increased the pancreatic insulin content of recovered degranulated β-cells, and even improved the β-cell secretory function. Islet vasculature remodeling has been shown to improve endocrine function and to be required for β-cells to thrive. Col IVα1 and laminin are the major constituents of all basement membranes. Within the islets, the basement membrane is found exclusively around the islet capillaries, but not islet cells [48]. Importantly, formation of intra-islet basement membrane seems to require the recruitment and retention of endothelial cells through VEGF-dependent mechanisms [49]. Col IV α1 and α2 as well as the laminin chains α4 and α5 act as signals from the islet basement membrane, which increase β-cell proliferation and insulin gene expression. Several researchers have already investigated the role of VEGF and SDF-1 in peripheral vasculogenesis and tissue damage repair. SDF-1 and its receptor chemokine receptor type 4 (CXCR4) have been attributed critical roles in (stem) cell recruitment to ischemic tissues [42]. There is increasing evidence that SDF-1 is an endogenous DPP-4 substrate and that almost all SDF-1 is inactivated *in vivo* by CD26/DPP-4-mediated cleavage. Incubation of intact SDF-1 in sera from CD26/DPP-4 knockout mice corresponded to the NH2-terminally intact form (1–67), whereas in sera from wild-type control mice it was generally converted to the inactive NH2-terminally truncated form (3-67)[19]. Here, we found that the combination therapy of anti-CD3+MK626 increased the expression of VEGF and SDF-1 locally in the pancreas. We speculate that under tight control of islet inflammation, MK626 as add-on to anti-CD3 therapy can create a microenvironment appropriate for the regranulation/repair of existing β-cells, leading to the restoration of the intra-islet basement membrane, and the maintenance of optimal β-cell secretory function.

These pre-clinical observations imply that DDP-4 inhibitors in combination with CD3 monoclonal antibodies can retain endogenous β-cell function and mass via direct effects on the islet cells but also via modulation of the pathogenetic autoimmune process and restoration of the islet microvasculature in turn favoring β-cell regranulation/repair. Moreover, these data provide evidence in support of clinical trials investigating short courses of anti-CD3/DPP-4 inhibitor combination therapy given early in the course of type 1 diabetes with the purpose of inducing long-lasting disease remission.

## Supporting Information

Figure S1 **Anti-CD3+MK626 treatment induced stable normoglycemia without body weight alterations in new-onset diabetic NOD mice.** (A) Random blood glucose values before and after treatment initiation. The left graph includes all mice. The right graph includes only mice with diabetes remission after therapy stop. (B) Glycemia curves from mice treated with anti-CD3+placebo (upper panel), and anti-CD3+MK626 (lower panel) with or without remission. (C) Weight change from recovered mice 2 and 7 weeks after anti-CD3+placebo or anti-CD3+MK626 therapy. Each dot represents an individual mouse. * vs. untreated new-onset diabetic mice.
(TIF)

Figure S2 **Levels of CD26 antigens in naïve, effector, memory and regulatory T cells after anti-CD3+MK626 therapy.** Frequency of CD26hi cells within naïve (CD44loCD69-CD62L+), effector (CD44hiCD69+CD62L-) and memory (CD44hiCD69-CD62L+) CD4+ (A) and CD8+ (B) T cell subsets from spleens and PLN of new-onset diabetic NOD mice that remained protected for 2 weeks following each of the treatments. (C) Frequency of CD26hi and CD26low cells within the CD4+CD25+FoxP3+ regulatory T cell population in spleens and PLN of new-onset diabetic NOD mice that remained protected for 2 weeks following each of the treatments. Each dot represents an individual mouse. * vs. new-onset diabetic mice. One symbol p<0.05; two symbols p<0.01.
(TIF)
