# Supplementary figures and images for: Combining MK626, a Novel DPP-4 Inhibitor, and Low-Dose Monoclonal CD3 Antibody for Stable Remission of New-Onset Diabetes in Mice
Source: PLoS One. 2014 Sep 30;9(9):e107935. doi: 10.1371/journal.pone.0107935 (PMC4182446; doi:10.1371/journal.pone.0107935)

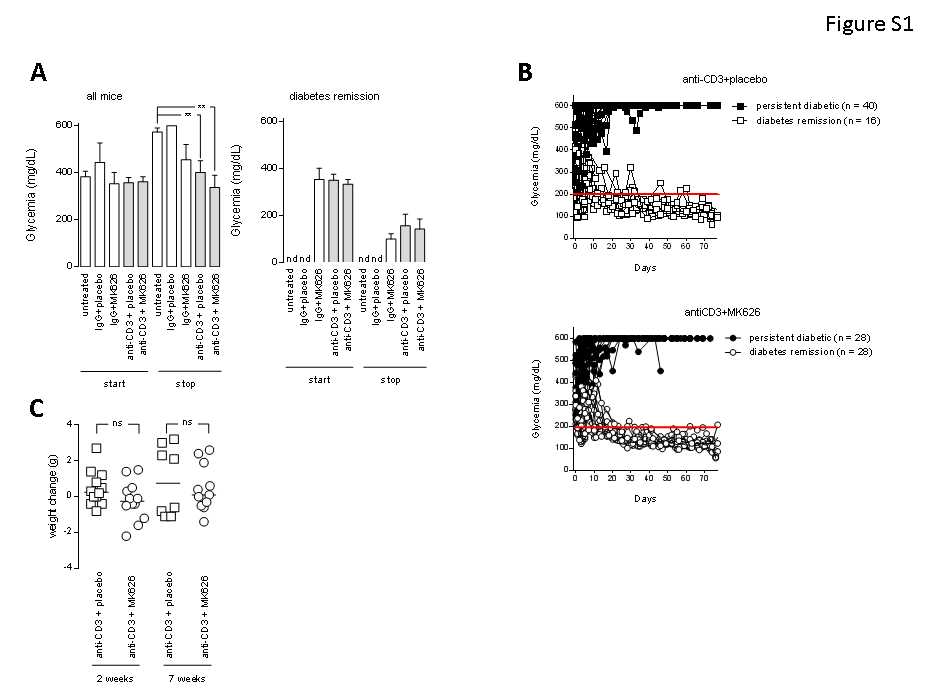

Supplement: Figure S1 — Anti-CD3+MK626 treatment induced stable normoglycemia without body weight alterations in new-onset diabetic NOD mice. (A) Random blood glucose values before and after treatment initiation. The left graph includes all mice. The right graph includes only mice with diabetes remission after therapy stop. (B) Glycemia curves from mice treated with anti-CD3+placebo (upper panel), and anti-CD3+MK626 (lower panel) with or without remission. (C) Weight change from recovered mice 2 and 7 weeks after anti-CD3+placebo or anti-CD3+MK626 therapy. Each dot represents an individual mouse. * vs. untreated new-onset diabetic mice. (TIF) [file pone.0107935.s001.tif]

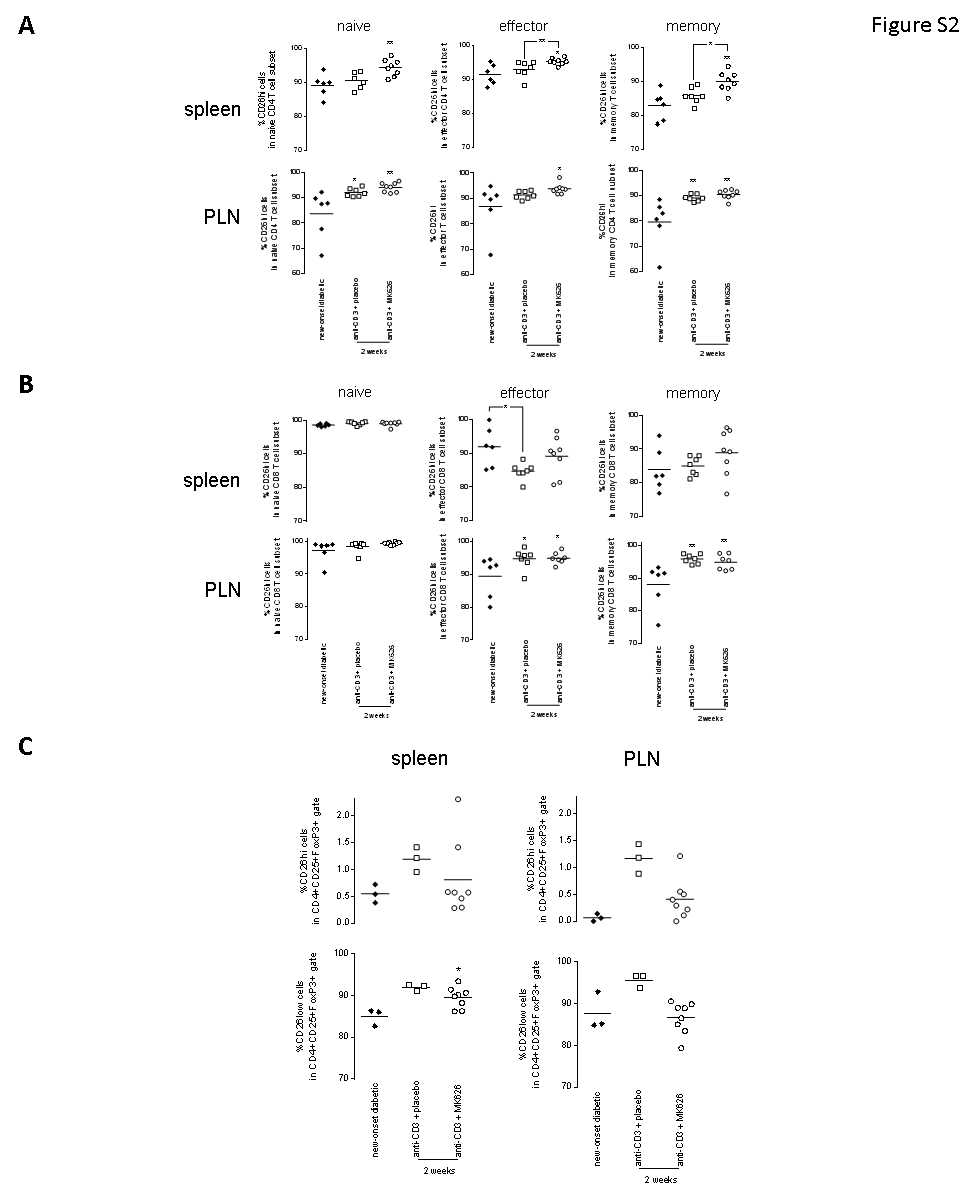

Supplement: Figure S2 — Levels of CD26 antigens in naïve, effector, memory and regulatory T cells after anti-CD3+MK626 therapy. Frequency of CD26hi cells within naïve (CD44loCD69-CD62L+), effector (CD44hiCD69+CD62L-) and memory (CD44hiCD69-CD62L+) CD4+ (A) and CD8+ (B) T cell subsets from spleens and PLN of new-onset diabetic NOD mice that remained protected for 2 weeks following each of the treatments. (C) Frequency of CD26hi and CD26low cells within the CD4+CD25+FoxP3+ regulatory T cell population in spleens and PLN of new-onset diabetic NOD mice that remained protected for 2 weeks following each of the treatments. Each dot represents an individual mouse. * vs. new-onset diabetic mice. One symbol p<0.05; two symbols p<0.01. (TIF) [file pone.0107935.s002.tif]
